# JAK/STAT signaling and human *in vitro *myogenesis

**DOI:** 10.1186/1472-6793-11-6

**Published:** 2011-03-09

**Authors:** Marissa K Trenerry, Paul A Della Gatta, David Cameron-Smith

**Affiliations:** 1 Molecular Nutrition Unit, Deakin University, Burwood, Victoria, Australia

## Abstract

**Background:**

A population of satellite cells exists in skeletal muscle. These cells are thought to be primarily responsible for postnatal muscle growth and injury-induced muscle regeneration. The Janus kinase/signal transducers and activators of transcription (JAK/STAT) signaling cascade has a crucial role in regulating myogenesis. In rodent skeletal muscle, STAT3 is essential for satellite cell migration and myogenic differentiation, regulating the expression of myogenic factors. The aim of the present study was to investigate and compare the expression profile of JAK/STAT family members, using cultured primary human skeletal muscle cells.

**Results:**

Near confluent proliferating myoblasts were induced to differentiate for 1, 5 or 10 days. During these developmental stages, members of the JAK/STAT family were examined, along with factors known to regulate myogenesis. We demonstrate the phosphorylation of JAK1 and STAT1 only during myoblast proliferation, while JAK2 and STAT3 phosphorylation increases during differentiation. These increases were correlated with the upregulation of genes associated with muscle maturation and hypertrophy.

**Conclusions:**

Taken together, these results provide insight into JAK/STAT signaling in human skeletal muscle development, and confirm recent observations in rodents.

## Background

Muscle fibres are terminally differentiated; therefore a population of quiescent satellite cells exists. These cells are thought to be responsible for postnatal muscle growth and injury-induced muscle regeneration [1]. Satellite cells are undifferentiated mononuclear cells, located within the basal lamina of the muscle fibre and reside in a dormant state until they are activated by physical activity or injury [2,3]. Upon activation they re-enter the cell cycle. Proliferating myoblasts expand their cytoplasmic-nuclei ratio and begin to fuse to existing fibres or with themselves to initiate de novo myofibre synthesis [3]. Muscle development is critically dependent on a family of myogenic regulatory factors (MRFs) including MyoD, Myf5, Myf6 and myogenin. They are temporally expressed to regulate the proliferation and differentiation of myoblasts, and often display overlapping roles. MyoD and Myf5 are expressed in actively proliferating cells prior to differentiation, while the expression of myogenin and Myf6 indicates that myoblasts have irreversibly withdrawn from the cell cycle and have commenced differentiation [4-7].

Numerous intracellular signaling pathways and molecules have been found to play several roles in myogenic differentiation. These include MAPK and ERK, which elicit different signals to promote or inhibit differentiation and fusion [8-13]. In addition, PI3K/Akt is utilized by IGF to stimulate differentiation while other growth factors such as HGF and FGF-2 enhance proliferation [8,14,15]. The JAK/STAT signaling cascade also appears to be an integral factor for myoblast development, known to be activated by IL-6 and LIF [16-21]. JAK family members, JAK1 and JAK2, are the most commonly used non-receptor tyrosine kinases. Seven STAT members exist, STAT1-4, STAT5a, 5b and 6, yet STAT3 was the first to be implicated in proliferation *in vitro *[14,19] and *in vivo *[21]. Recently in rodent models, specific roles have been defined for several JAK and STAT members. It was demonstrated that JAK1/STAT1/STAT3 signaling is involved in myoblast proliferation preventing premature differentiation [16]. However, JAK2/STAT2/STAT3 appears to positively regulate differentiation indicating that STAT3 elicits specific responses at various times during myogenesis [22].

The temporal responsiveness of JAK/STAT signaling in humans is largely unknown. Therefore, the aim of this study was to investigate the expression of JAK/STAT signaling molecules *in vitro *during human myoblast differentiation. Near confluent proliferating myoblasts were induced to differentiate for 1, 5 or 10 days. During these developmental stages, members of the JAK/STAT family were examined, along with factors known to regulate myogenesis. It was hypothesized that STAT3 signaling would be elevated during myogenesis as it is essential for both proliferation and differentiation in murine cells, and we expected STAT1 phosphorylation to be restricted to proliferation.

## Results

### Myoblasts undergoing differentiation display a typical genetic profile

**Figure 1:**
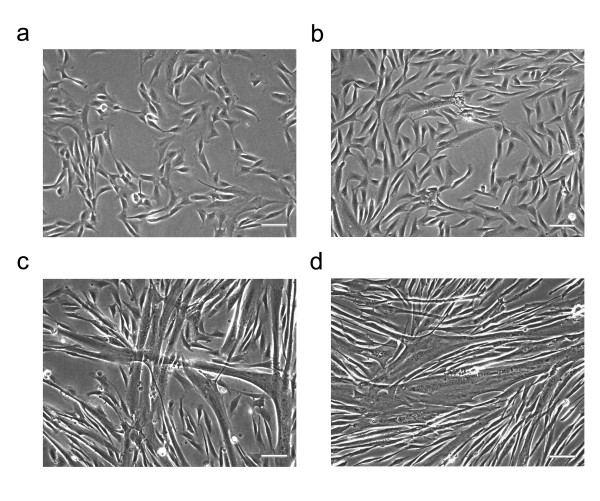
**Phase contrast images of developing human skeletal muscle cells**. Primary myoblasts were grown to near confluence (a) and induced to differentiate for 1 (b), 5 (c) and 10 (d) days. Scale bar represents 50 μm.

Phenotypically, the cells used in these experiments were successfully undergoing differentiation (Figure 1). Proliferating cells demonstrated a high nuclei-cytoplasmic ratio (Figure 1a). Serum depletion initiated differentiation, where myoblasts became elongated (Figure 1b) and fused with nearby cells to form multinucleated tubes (Figure 1c). Following 10 days of serum depletion, large myotubes were evident with a low number of single nuclei myoblasts remaining (Figure 1d).

**Figure 2:**
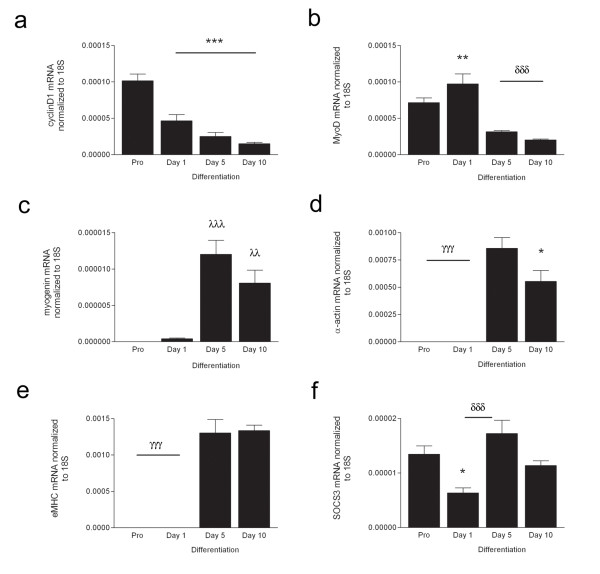
**Genes associated with myogenesis are differentially regulated during myoblast differentiation**. The expression of *cyclinD1 *(a), *MyoD *(b), *myogenin *(c), *α-actin *(d), *eMHC *(e) and *SOCS3 *(f) mRNA was measured in primary human myoblasts. Values are arbitrary units normalized to the expression levels of the reference gene *18 S *representing the mean of 6 replicates ± SEM. Significantly different from Pro: * p < 0.05, ** p < 0.01, *** p < 0.001. Significantly different from Day 1: δδδ p < 0.001. Significantly different from Pro and Day 1: λλ p < 0.01, λλλ p < 0.001. Significantly different from Day 5 and Day 10: γγγ p < 0.001.

To confirm these observations, the mRNA expression of genes known to be involved in myogenesis were investigated; *cyclinD1 *(a), *MyoD *(b), *myogenin *(c), *α-actin *(d), *eMHC *(e) and *SOCS3 *(f) (Figure 2). *cyclinD1 *was significantly decreased following the initiation of differentiation (p < 0.05). *MyoD *expression increased 1.5-fold at the onset of differentiation (Day 1) (p < 0.01). By Day 5 and 10, *MyoD *was below the level observed in proliferating cells (p < 0.001). *Myogenin *expression was 870- and 585-fold higher (Day 5 and 10 respectively) than that seen in proliferating cells (p < 0.001, p < 0.01 respectively). *α-actin *and *eMHC *were measured as markers of myotube growth. *α-actin *and *eMHC *were significantly higher in differentiating myoblasts compared to both proliferating and Day 1 cells (p < 0.001). Finally, *SOCS3 *mRNA expression displayed a significant decrease at the onset of differentiation (p < 0.05), however this was again elevated by Day 5 of differentiation (p < 0.001), thereafter it returned to the level observed during proliferation.

### Elements of STAT signaling are differentially expressed during myogenesis

**Figure 3:**
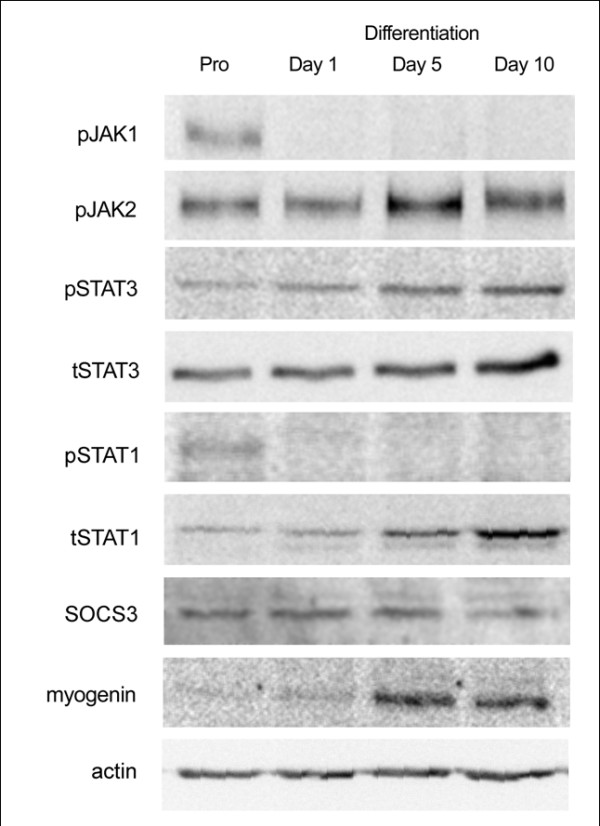
**Temporal expression of JAK/STAT during myogenic differentiation in human muscle cells**. Representative Western blots of protein extracted from near confluent primary human myoblasts that were induced to differentiate during the specified times. Phosphorylation of JAK2 (Tyr1007/1008) and STAT3 (Tyr705) increases during differentiation, while JAK1 (Tyr1022/1023) and STAT1 (Tyr701) were only apparent during proliferation.

**Figure 4:**
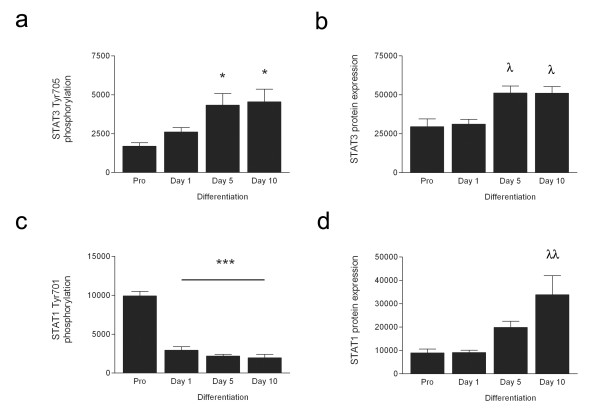
**STAT3 and STAT1 are differentially expressed during myogenic differentiation in human muscle cells**. Graphs represent phosphorylated and endogenous levels of STAT3 (a, b) and STAT1 (c, d) in protein extracted from near confluent primary human myoblasts that were induced to differentiate during the specified times. Values are arbitrary units representing the mean of 6 replicates ± SEM. Significantly different from Pro: * p < 0.05, *** p < 0.001. Significantly different from Pro and Day 1: λ p < 0.05, λλ p < 0.01.

To investigate STAT signaling during human myoblast differentiation, we measured the phosphorylation of STAT family members STAT1 and STAT3 as well as the negative regulator, SOCS3. Tyr705 phosphorylation increased during myoblast differentiation (p < 0.05; proliferating cells compared to day 5 and day 10), which was accompanied by a concomitant increase in total STAT3 (p < 0.05; proliferating cells compared to day 5 and day 10). Similarly there was an increase in total STAT1 during differentiation (p < 0.01); however phosphorylation of Tyr701 was only evident in proliferating myoblasts (Figure 3 and 4). Interestingly, unlike SOCS3 mRNA, SOCS3 protein levels remained unchanged during myoblast development (Figure 3). Phosphorylation of upstream kinases, JAK1 and JAK2 was measured; JAK1 phosphorylation at Tyr1022/1023 was only evident in proliferating cells while Tyr1007/1008 phosphorylation of JAK2 appeared to increase during differentiation. To confirm the commitment of myoblasts to differentiation, myogenin protein expression was also measured; this was only evident in Day 5 and Day 10 cells. Actin was used as a loading control (Figure 3).

## Discussion

Due to the complexity of intracellular signaling, the molecular mechanisms underlying skeletal muscle development remain partially understood. It is widely accepted that myogenesis is critically dependent on MRFs, however numerous other signaling pathways have been found to be influential on myogenic differentiation. Therefore, it is important to investigate the signaling pathways that may be involved in the structural remodeling of skeletal muscle. JAK/STAT is now recognized as a critical pathway needed for efficient muscle fibre adaptation. In murine cell models, it was identified that the JAK1/STAT1/STAT3 axis is involved in myoblast proliferation preventing the premature differentiation into myotubes [16]. However, it is JAK2/STAT2/STAT3 that appears to positively regulate differentiation, indicating that STAT3 elicits specific responses at various times during myogenesis [22], possibly via JAK/STAT co-operating with various ligands to initiate distinct cellular responses.

Primary human muscle cells provide an appropriate experimental model that has human physiologic relevance. Therefore, using primary human skeletal myoblasts, we sought to corroborate the results obtained in murine cells [16,17,22]. Near confluent myoblasts were exposed to low serum media to induce differentiation for 1, 5 or 10 days. The expression profile of JAK/STAT family members was investigated in conjunction with factors known to regulate myogenesis. As the myoblasts were undergoing differentiation, they displayed a typical genetic expression profile. MRFs are temporally expressed to regulate the proliferation and differentiation of myoblasts. MyoD is expressed in actively proliferating cells prior to differentiation, followed by increased myogenin expression at the beginning of differentiation [4-6]. At the onset of differentiation there is also a withdrawal from the cell cycle, represented by changes in expression of cell cycle regulators. As myofibres mature, they begin to fuse to form multinucleated muscle cells which is accompanied by the expression of MHC and α-actin [7,23]. In primary human skeletal myoblasts, STAT3 Tyr705 phosphorylation increased during myoblast differentiation, which was accompanied by an apparent elevation in endogenous STAT3, indicating a role during both the proliferative and differentiation phases. STAT3 elevations were associated with unchanged SOCS3 protein expression during the time course. However, SOCS3 mRNA expression was significantly lower at Day 1 of differentiation compared to proliferating and Day 5 differentiated muscle cells, consistent with earlier studies [24]; further highlighting the importance of STAT3 signaling during the onset of differentiation.

Unlike STAT3, STAT1 Tyr701 phosphorylation was only evident during proliferation despite endogenous STAT1 protein increasing during differentiation. In murine cells, it has been demonstrated that STAT1 is important for proliferation, therefore it was expected that we would observe STAT1 activity only during this stage. However, it has been described that STAT1 activation can often reduce proliferation [25]. Accordingly, it may be reasonable to assume that STAT1 phosphorylation during proliferation occurs when it complexes with STAT3 to prevent myoblast differentiation [16]; yet when cells undergo differentiation, STAT1 may be acting alone to inhibit myoblast proliferation [25].

Upstream of STATS are gp130, and non-receptor tyrosine kinases, JAK1 and JAK2. Previously it has been demonstrated that JAK1 and JAK2 have differential roles during murine myoblast differentiation despite their high homology; JAK1 is essential for proliferation, while JAK2 is necessary for differentiation [16,22]. In this study, JAK1 phosphorylation was only present in proliferating myoblasts; this is consistent with its role in proliferation as described in rodent models [16]. JAK2 was phosphorylated during each time point, which was unexpected given its reported role in differentiation [22]. However, this may indicate that there was a population of myoblasts that were spontaneously differentiating, in the proliferating sample. This may also account for the higher than expected expression of *MyoD *observed in the proliferating cells.

## Conclusions

The current study demonstrates that STAT signaling during myogenesis is similar in humans as to what has been described in rodents. Importantly, as STAT3 plays an integral role in myoblast maturation and skeletal muscle adaptation, we observed an increase in STAT3 Tyr705 phosphorylation during differentiation which was accompanied by an apparent elevation in endogenous STAT3. Although our results are similar to that demonstrated in rodents, our observations alone should not be used to definitively identify the role of JAK/STAT signaling in human skeletal myogenesis; further mechanistic investigation is warranted to clearly define the importance of this pathway in human skeletal muscle development, similar to that which has been performed in murine models [16,17,20,22,24]. Additionally, as this pathway is critical for myogenesis, it may be an appropriate therapeutic target for diseases with impaired muscular adaptation and regeneration.

## Methods

### Primary Skeletal Muscle Cell Culture

Primary skeletal muscle cell culture was established according to previously described methods [26,27]. Skeletal muscle samples were excised using the percutaneous needle biopsy technique [28] modified to include suction [29] from the *vastus lateralis *of healthy young males, in accordance with the Deakin University Ethics Committee, where written and verbal informed consent was obtained. The excised muscle was immersed and extensively washed in ice-cold Hams F-10 medium (Invitrogen, Melbourne, Australia) before being minced in ice-cold Hams F-10 medium (Invitrogen, Melbourne, Australia). Minced tissue was then digested in 25 ml 0.05% Trypsin/EDTA (Invitrogen, Melbourne, Australia) at 37°C with agitation for 20 min to release the myoblasts. The supernatant containing the myoblasts was then collected and the process repeated a further two times to breakdown any remaining tissue. Horse serum (HS) (Invitrogen, Melbourne, Australia) was subsequently added to the supernatant to a final concentration of 10%. The supernatant was filtered through a pre-wet 74 μm (15 mm diameter) filter (Sigma-Aldrich, Sydney, Australia) to remove any connective tissue and then centrifuged for 10 min at 1600 rpm to collect the cells. The resulting cell pellet was re-suspended in Hams F-10 medium containing 20% Fetal Bovine Serum (FBS) (Invitrogen, Melbourne, Australia) with 25 ng/ml bFGF (Invitrogen, Melbourne, Australia), 0.05% pen/strep (Invitrogen, Melbourne, Australia) and 0.05% amphoterecin (Invitrogen, Melbourne, Australia). The cells were then seeded on to an uncoated 25 cm^2 ^flask and incubated at 37°C for 30 min to induce fibroblast attachment, leaving myoblasts suspended in the medium. The medium was aspirated and this process was repeated for another 30 min. The medium was aspirated and seeded on to an extracellular matrix (ECM) (Sigma-Aldrich, Sydney, Australia) coated 25 cm^2 ^flask. The resulting primary cell cultures were maintained in F10 Nutrient Mixture (Invitrogen, Melbourne, Australia) containing 20% FBS with 25 ng/ml bFGF, 0.05% pen/strep and 0.05% amphoterecin in humidified air at 37°C and 5% CO_2_.

Cells were washed twice with PBS and detached from the flask surface using TrypLE™ (Invitrogen, Melbourne, Australia) and seeded onto ECM coated petri dishes (protein) and 6 well plates (RNA). When cells reached 70% confluence they were either left to proliferate in growth media, or induced to differentiate with DMEM/F12 (Invitrogen, Melbourne, Australia) containing 2% HS, 0.05% pen/strep and 0.05% amphoterecin for 1, 5 or 10 days. Images were captured with an Olympus IX51 (Olympus, Australia) using AnalySIS getIT software (Olympus, Australia).

### Protein Extraction and Western Blot Analysis

Cells were washed twice with PBS followed by the addition of RIPA lysis buffer (Millipore, Billerica, MA). The lysate was rotated at 4°C for 1 h then centrifuged at 13000 rpm at 4°C for 10 min and the supernatant collected for analysis of protein concentration (BCA protein assay kit, Pierce Biotechnology, Rockford, IL). Protein samples (20 μg) were denatured in sample buffer and separated by 8% SDS-PAGE. The proteins were transferred onto a PVDF membrane and soaked in methanol for 2 min then left to air dry for 20 min [30]. Primary antibodies, pSTAT3, tSTAT3, pSTAT1, tSTAT1, pJAK1 and pJAK2 (Cell Signaling, Danvers, MA) diluted in 5% BSA/TBST; SOCS3 (H103) (Santa Cruz Biotechnology, Santa Cruz, CA) diluted in 5% skim milk/PBST; myogenin (Santa Cruz Biotechnology, Santa Cruz, CA) diluted in 5% skim milk/TBST and actin (Sigma-Aldrich, Sydney, Australia) diluted in 4% cold fish gelatin (CFG)/TBST were applied and incubated overnight at 4°C. Membranes were subsequently washed with TBST and incubated for 1 h at room temperature with HRP-conjugated secondary antibodies before being washed again. Proteins were visualized by enhanced chemiluminescence (Western Lighting Chemiluminescence Reagent Plus, Perkin-Elmer, Boston, MA). The density of the bands were quantified using Kodak Imaging software, Kodak ID 3.5 (Perkin Elmer Life Sciences, Boston, MA).

### RNA Extraction and RT-PCR

Cells were washed twice with PBS followed by the addition of TRI-Reagent (Applied Biosystems, Foster City, CA). Chloroform (Sigma-Aldrich, Sydney, Australia) was added to separate the phases. Following centrifugation, the aqueous layer was removed, an equal volume of isopropanol (Sigma-Aldrich, Sydney, Australia) was added and the RNA precipitated at -20°C for 2 h. The RNA was centrifuged at 13000 rpm to pellet the RNA. The pellet was washed with 75% ethanol and then re-suspended in nuclease free water. RNA quality and concentration were determined using the NanoDrop 1000 Spectrophotometer (Thermo Scientific, Australia). First-strand cDNA was generated from 0.5 μg total RNA using High Capacity RNA-to-cDNA kit (Applied Biosystems, Foster City, CA). RT-PCR was performed using the Applied Biosystems 7500 Real Time PCR System (Applied Biosystems, Foster City, CA). PCR was performed in duplicate with reaction volumes of 20 μl, containing Power SYBR Green 1 (Applied Biosystems, Foster City, CA), forward and reverse primers and cDNA template (diluted 1:20). Data were analyzed using a comparative critical threshold (Ct) method where the amount of target normalized to the amount of endogenous control relative to control value is given by 2^-ΔΔCt^. The efficacy of *18 S *as an endogenous control was examined using the equation 2^-ΔCt^. No change in the expression of this gene was observed (data not shown) so it was considered an appropriate endogenous control for this study. Primers are outlined in Table 1.

**Table 1:** Details of primers used for real-time PCR analysis

Gene	GenBank Accession Number	Forward Primer (5'-3')	Reverse Primer (5'-3')
*18S*	NR_003286	TTCGGACGTCTGCCCTATCAA	ATGGTAGGCACGGCGACTA
*α-actin*	NM_001100	GTAGCTAACCGCCCAGAAACT	AGGCCGGAGCCATTGTC
*CyclinD1*	NM_053056	GCATGTTCGTGGCCTCTAAGA	CGGTGTAGATGCACAGCTTCTC
*eMHC*	NM_002470	TCCTGGCTGTTGCTGTCTTCT	ACTTCCATTTCAGTGTCACTACTCATG
*MyoD*	NM_002478	CCGCCTGAGCAAAGTAAATGA	GCAACCGCTGGTTTGGATT
*Myogenin*	NM_002479	GGTGCCCAGCGAATG	TGATGCTGTCCACGATCGA
*SOCS3*	NM_003955	GACCAGCGCCACTTCTTCA	CTGGATGCGCAGGTTCTTG

Primer sequences were designed using Primer Express version 3.0 software (Applied Biosystems) using sequences accessed through GenBank and checked for specificity using Nucleotide-Nucleotide Blast search. Embryonic myosin heavy chain, eMHC.

### Statistical Analysis

Statistical analysis was performed using SPSS 15.0. Unless stated otherwise, means were compared using a one-way ANOVA and significant differences were determined using a Bonferroni Post Hoc Test. Data is presented as mean ± SEM. A probability level of <0.05 was adopted throughout to determine statistical significance unless otherwise stated.

## Authors' contributions

MT carried out the experiments, performed the statistical analysis and drafted the manuscript. PDG participated in the development of the cell culture model. DCS conceived of the study, and participated in its design and coordination and helped to draft the manuscript. All authors read and approved the final manuscript
